# Economic tolerance design of the P2 raceway based on the quasi-static model of aerospace ball bearings

**DOI:** 10.1038/s41598-024-54401-5

**Published:** 2024-02-13

**Authors:** Zhou Chang, Lai Hu, Wenhan Cao

**Affiliations:** 1 School of Mechanical and Electrical Engineering, Lanzhou Jiaotong University, Lanzhou, 730070 Gansu People’s Republic of China; 2 State Key Laboratory for Manufacturing System Engineering, Xi’an Jiaotong University, Xi’an, 710054 Shaanxi People’s Republic of China

**Keywords:** Tolerance, Economic design, P2 Raceway, Quasi-static model, Ball bearing, Aerospace engineering, Mechanical engineering

## Abstract

This study focused on designing the raceway tolerance of high-precision rolling bearings. After the tolerance error equivalence relationship is defined, the application of the rolling bearing error equalization effect in tolerance design is studied. First, a five-degree of freedom quasi-static model was established for angular contact ball bearings. The waviness was included in the manufacturing error, and the radial and axial runouts of the bearing inner ring were calculated. Second, the error homogenization effect of the rolling bearing was studied, and the error homogenization coefficient was defined. The results of the study demonstrated that the bearing rotary accuracy was higher than the raceway error by an order of magnitude. Third, the manufacturing error range of each precision grade of the rolling bearing raceway was obtained by investigating errors of the equalization coefficient. Finally, the specific value of the raceway tolerance of P2 rolling bearings was obtained.

## Introduction

Studies have shown that high-speed and heavy-load ball bearings should have a stable and precise long-term operation^1^; hence, investigation of the runout of precision bearings is an important topic in research on the bearing dynamic problem. Various theoretical models have been proposed to date for the runout of rolling bearings. These models can be divided into four categories: static, quasi-static, dynamic, and pseudo-dynamic models^1^.

In 1960, Jones et al*.*^2^ proposed a quasi-static model for ball bearings. In this model, centrifugal forces and gyroscopic moment of the rolling element are considered to construct torque balance equations between the ring and the steel ball. Then, the resulting equations are solved through an appropriate numerical method. Moreover, the theory of raceway control was applied to analyze the dynamics of the rolling bearing. Based on the concept of elastohydrodynamic lubrication (EHL), Harris et al*.* established a quasi-static method to analyze high-speed ball bearings and then improved the performance of the model using an empirical drag force expression instead of the raceway control theory^3,4^. Furthermore, Harris et al*.* introduced the EHL effect on the Jones quasi-static model, considered the inertia effect of the rolling element, comprehensively analyzed the cage influence of the rolling element and ring, and established a pseudo-dynamic model of the ball bearing^5,6^.

Walters et al*.*^7,8^ established a dynamic model for ball bearings, which laid the foundation of the dynamic model for rolling bearings. Then, Walters’ model was applied to develop the BASDAP software for analyzing rolling bearings. In the proposed dynamic model, different aspects of Walters’ dynamic model are considered. Moreover, Walters’ model comprehensively analyzes the interaction between the steel ball and raceway, steel ball and cage, and cage and ferrule, thereby establishing the motion differential equations. An appropriate numerical integration can be applied based on initial conditions to solve the resulting nonlinear equations. Based on the Walters’ model, Gupta et al*.*^9–12^ analyzed the transient and steady-state dynamic characteristics of the rolling bearing and then established the dynamic model of the ball bearing. The main hypothesis of the Gupta’s model is that the contact between the steel ball and the cage is an elastic collision, and thus, it does not consider the lubrication effect on the bearing performance. The ADORE software was developed based on the Gupta’s kinetic model, and it can be applied in most of the bearing dynamic problems. However, the calculation expenses in complicated problems cannot be justified.

A review of the literature of analysis of bearing dynamics shows that obtaining an in-depth understanding of nonlinear collision problems has become a research focus. Although bearing dynamics has been studied in detail, the simulation expenses of current methods are very high. To address this shortcoming, the quasi-static calculation method and EHL theory were used in this study, and the error homogenization effect of the rolling bearing was studied. Chang Zhou's paper on this subject has been published online in Preprints^15^. This paper has now been revised with some additions.

The main objective of this study was to provide a theoretical basis for the economic design of bearing raceway tolerance. The obtained results are expected to contribute to the body of knowledge on economic tolerance design.

## Introduction of tolerance error equivalence relationship

There is an equivalent relationship between tolerance and error, i.e. the size and distribution of tolerance will directly affect the error situation of the product. Tolerance is the allowable range of deviation between the design size and the actual size, while error is the deviation between the actual size and the design size.

The purpose of tolerance design is to control the error of the product within a certain range to ensure that the function and quality requirements of the product are met. Therefore, tolerance design needs to take into account the functional requirements of the product, the limitations of the machining process and the cost.

The common method used in tolerance design is to determine the size and distribution of tolerances by statistical methods. By measuring and analyzing the dimensions of the product, the error distribution of the product can be obtained. Then, depending on the functional requirements of the product and the limitations of the manufacturing process, the appropriate tolerance range and distribution are determined.

In addition, mathematical modeling and simulation methods can be used to investigate the relationship between tolerances and errors. By building mathematical models and performing simulation analysis, the error situation of the product can be predicted and the tolerance design can be optimized.

In conclusion, there is an equivalent relationship between tolerance and error, and the size and distribution of tolerance will directly affect the error situation of the product. Tolerance design research aims to minimize the error while meeting the functional and quality requirements of the product through reasonable tolerance distribution and control.

## Establishment of 5-DOF quasi-static model

Each rolling bearing has three translational and two rotational degrees of freedom (DOF). In this section, a 5-DOF quasi-static model of the rolling bearing is introduced based on the mathematical correlation between the applied load on the bearing and the corresponding displacement of the inner ring.

**Figure 1:**
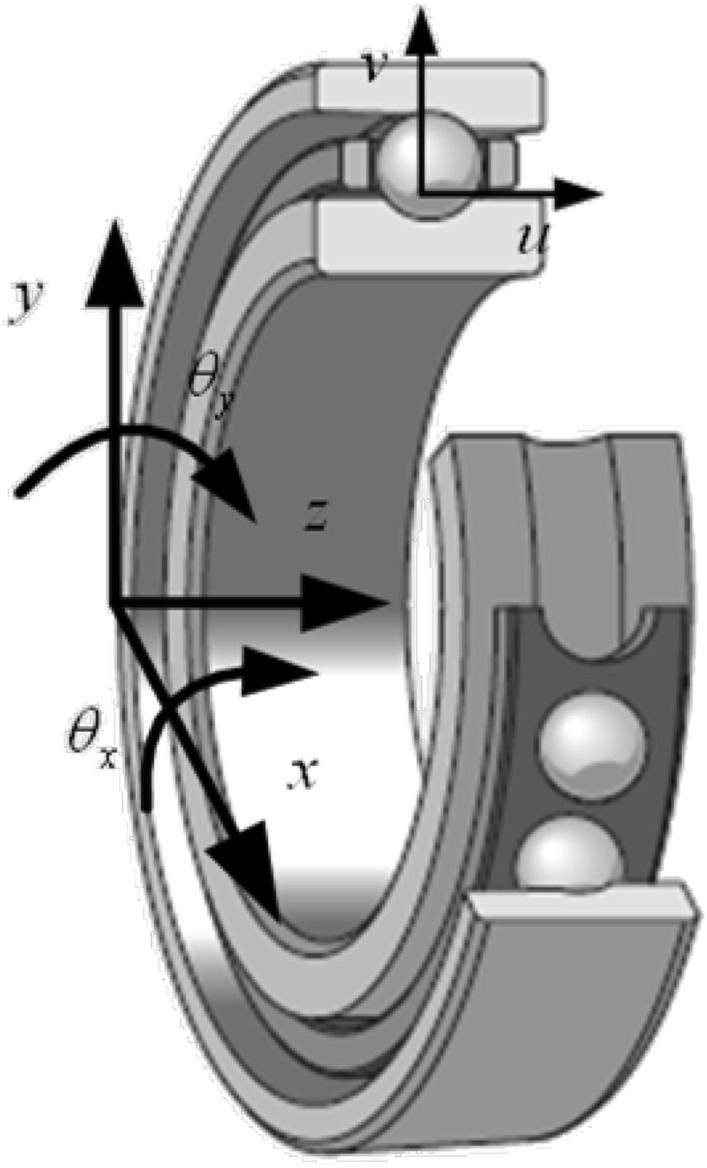
Coordinate systems in the ball bearing.

To facilitate analysis and numerical calculations, two coordinate systems were established as shown in Fig. 1:

1. the global coordinate system $(x,y,z)$, the origin is located at the center of the inner ring of the bearing. The x-axis and y-axis are two mutually perpendicular radial directions of the bearing and are located in the center plane of the bearing collar, and the z-axis coincides with the center of rotation of the bearing.
2. the local coordinate system $(u,v)$, the origin is located at the center of curvature of the outer ring channel. u-axis is parallel to z-axis and v-axis is in the same radial direction as the bearing.

In the global coordinate system, the displacement of the inner ring can be expressed as ($x$,$y$,$z$,$\theta_{x}$,$\theta_{y}$). Considering the raceway waviness error, the displacement of the center of the *j*th steel ball in the local coordinate system can be expressed as follows:

1 $$ v_{j} = x\cos \psi_{j} + y\sin \psi_{j} - q_{ij} \theta_{x} \sin \psi_{j} + q_{oj} \theta_{y} \cos \psi_{j} + (p_{ij} - p_{oj} ), $$

2 $$ u_{j} = z + (\Re_{i} + p_{ij} )\theta_{x} \sin \psi_{j} - (r_{p} + p_{ij} )\theta_{y} \cos \psi_{j} + q_{ij} , $$

where $\psi_{j}$ is the position angle of the $j$ ^th^ ball; $q_{ij}$, $q_{oj}$,$p_{ij}$, and $p_{oj}$ denote the waviness value of the inner and outer rings at the position of the $j$th ball, and $\Re_{i}$ is the distance between the curvature center of the inner-ring raceway and the rotation center of the bearing. $\psi_{j}$ and $\Re_{i}$ can be expressed by Eqs. (3) and (4), respectively:

3 $$ \psi_{j} = 2\pi (j - 1)/Z + \psi_{0} + \omega_{i} t(1 - D_{b} \cos \alpha_{0} /d_{m} ), $$

4 $$ \Re_{i} = d_{m} /2 + (f_{i} - 0.5)D_{b} \cos \alpha_{0} , $$

where $\psi_{0}$ is the initial position angle of the first ball, which is generally set to zero.

**Figure 2:**
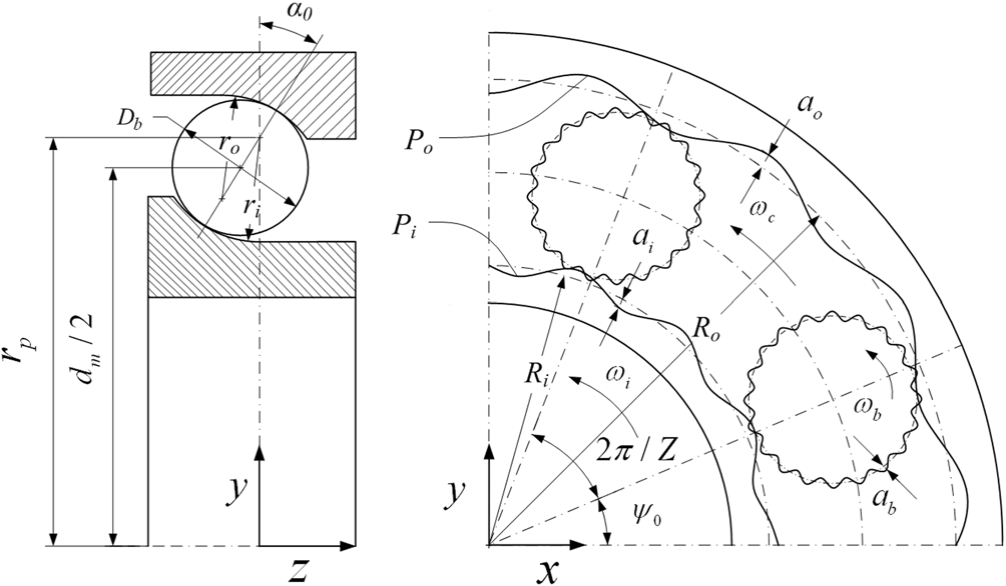
Geometric parameters and machining error of the ball and raceway.

Figure 2 illustrates the Geometric parameters and machining error of the ball and raceway.

### Roundness model

The waviness can be expressed in the form of harmonics; therefore, the radial waviness (roundness) of the inner and outer raceways of the bearing can be expressed by Eqs. (5) and (6) respectively:

5 $$ p_{ij} = \sum\limits_{n = 1}^{N} {a_{in} \cos (n(\psi_{j} - \omega_{i} t) + \phi_{in} )} , $$

6 $$ p_{oj} = \sum\limits_{n = 1}^{N} {a_{on} \cos (n\psi_{j} + \phi_{on} )} , $$

where $a_{in}$ and $a_{on}$ are the amplitude, and $\phi_{in}$ and $\phi_{on}$ are the phase of the radial waviness.

### Groove model

Similarly, the axial waviness (groove shape) of the inner- and outer-ring raceways of the bearing can be expressed by Eqs. (7) and (8) respectively:

7 $$ q_{ij} = \sum\limits_{n = 2}^{N} {b_{in} cos(n(\psi_{j} - \omega_{i} t) + \eta_{in} )} , $$

8 $$ q_{oj} = \sum\limits_{n = 2}^{N} {b_{on} \cos (n\psi_{j} + \eta_{on} )} , $$

where $b_{in}$ and $b_{on}$ denote the amplitude; $\eta_{in}$ and $\eta_{on}$ denote the phase of axial waviness.

### Side-sway model

The side-sway is the first-order Fourier expansion of the axial waviness with a groove shape. In this study, the inner- and outer-ring raceway side-sway are defined by Eqs. (9) and (10), respectively:

9 $$ C_{ij} = b_{i1} cos((\psi_{j} - \omega_{i} t) + \eta_{i1} ), $$

10 $$ C_{oj} = b_{o1} \cos (\psi_{j} + \eta_{o1} ). $$

### Sphericity model

Based on the contact characteristics of steel balls, the difference between the contact points of the steel ball and inner ring and outer ring is 180°. Therefore, the sphericity waviness at the contact points of the steel ball in the inner and outer rings can be expressed as follows:

11 $$ w_{ij} = \sum\limits_{n = 2}^{N} {a_{bnj} \cos (n\omega_{R} t + \lambda_{bnj} )} , $$

12 $$ w_{oj} = \sum\limits_{n = 2}^{N} {a_{bnj} \cos (n(\omega_{R} t + \pi ) + \lambda_{bnj} )} , $$

where $a_{bnj}$ and $\lambda_{bnj}$ denote the amplitude and phase of the order waviness on the $j$th ball, respectively.

### Deviation model of the ball diameter

In this study, the diameter deviation of the steel ball is modeled as the following:

13 $$ D_{bj} = D_{b} + \Delta D_{bj} , $$

where $\Delta D_{bj}$ is the diameter deviation of the $j$th ball. In general, the ISO-tolerance level of the steel ball is G5, indicating that the maximum diameter tolerance of the batch of steel balls is 0.25 μm, while the maximum amplitude of the first-order error of the steel ball waviness is 0.13 μm. Moreover, the elastic oil film thickness of the raceway and steel ball is generally approximately 1 μm, indicating that the calculation of steel ball error will not fracture the elastic oil films.

## Influence of the oil film on error homogenization

Hamrock and Dowson^14^ solved the EHL oil film thickness problem, obtaining pressure distributions of the point contact under isothermal conditions, and proposed an empirical expression for the oil film center thickness in the form below:

14 $$ h = 2.69R_{x} V^{\prime 0.69} G^{\prime 0.53} W^{\prime - 0.067} (1 - 0.061e^{ - 0.73\kappa } ). $$

Equation (14) indicates that although the oil film thickness depends on the contact load, the effective coefficient is only − 0.067. Therefore, in solving the quasi-static model of the ball bearing without considering the lubrication impact, the quasi-static model of ball bearing can be solved according to the following methods. First, thereby calculating the contact load. Then, the results obtained from Eq. (14) can be used as the iterative initial value for solving the oil film thickness. In this way, challenging problems such as those with difficult-to-determine initial values for oil film thickness can be solved accurately.

Despite the advent of diverse numerical methods, the Hertzian contact problem is still a challenging problem in the field of EHL. In this study, the derivative of contact load to the oil film thickness is used as the oil film stiffness^14^:

15 $$ k_{c} = - \frac{10}{{1.8023}}E^{\prime } R_{x} \left[ {V^{\prime 0.67} G^{0.53} W^{ - 1.067} (1 - 0.61e^{ - 0.73\kappa } )} \right]^{ - 1} . $$

## Solution of the quasi-static equation

Using the balance condition of the whole bearing, the following expressions can be obtained:

16 $$ F_{rx} = \sum\limits_{j = 1}^{Z} {(Q_{ij} cos\alpha_{ij} + \frac{{\lambda_{ij} M_{gj} }}{{D_{b} }}sin\alpha_{ij} } )cos\psi_{j} $$

17 $$ F_{ry} = \sum\limits_{j = 1}^{Z} {(Q_{ij} cos\alpha_{ij} + \frac{{\lambda_{ij} M_{gj} }}{{D_{b} }}sin\alpha_{ij} } )sin\psi_{j} $$

18 $$ F_{a} = \sum\limits_{j = 1}^{Z} {(Q_{ij} sin\alpha_{ij} - \frac{{\lambda_{ij} M_{gj} }}{{D_{b} }}cos\alpha_{ij} } ) $$

19 $$ M_{rx} = \sum\limits_{j = 1}^{Z} {\left[ { - q_{ij} \left( {Q_{ij} cos\alpha_{ij} + \frac{{\lambda_{ij} M_{gj} }}{{D_{b} }}sin\alpha_{ij} } \right) + \left( {Q_{ij} \sin \alpha_{ij} - \frac{{\lambda_{ij} M_{gj} }}{{D_{b} }}\cos \alpha_{ij} } \right)(\Re_{i} + p_{ij} ) + {\kern 1pt} \frac{{\lambda_{ij} M_{gj} }}{{D_{b} }}r_{i} } \right]\sin \psi_{j} } $$

20 $$ M_{ry} = \sum\limits_{j = 1}^{Z} {\left[ {q_{ij} \left( {Q_{ij} cos\alpha_{ij} + \frac{{\lambda_{ij} M_{gj} }}{{D_{b} }}sin\alpha_{ij} } \right) - \left( {Q_{ij} \sin \alpha_{ij} - \frac{{\lambda_{ij} M_{gj} }}{{D_{b} }}\cos \alpha_{ij} } \right)(\Re_{i} + p_{ij} ) - {\kern 1pt} {\kern 1pt} {\kern 1pt} {\kern 1pt} \frac{{\lambda_{ij} M_{gj} }}{{D_{b} }}r_{i} } \right]\cos \psi_{j} } $$

By solving these equations simultaneously, the displacements of the bearing in five directions, that is,$x$, $y$,$z$, $\theta_{x}$, and $\theta_{y}$, can be obtained.

## The solution method

The above equations can be solved according to the following methods. Each ball used for calculation has four unknown parameters, that is,$\delta_{ij}$,$\delta_{oj}$,$X_{1j}$, and $X_{2j}$. For example, 7014 series bearings contain 18 steel balls, thereby having 72 unknown parameters for steel balls. Meanwhile, the inner-ring displacement has five unknown parameters, namely,$x$, $y$, $z$, $\theta_{x}$, and $\theta_{y}$. Therefore, the bearing inner ring and steel ball have 72 + 5 unknowns, resulting in 77 nonlinear equations to be solved simultaneously.

Because the distance between the steel balls is small, the contact angle between the adjacent balls does not change much; therefore, the iterative contact angle of the previous ball can be considered as the initial contact angle of the next ball. The steel ball with the largest stress is selected as the breakthrough of the research object. The contact angle is defined as $\alpha_{i}$ and $\alpha_{o}$. Then appropriate initial values for $x$,$y$, $z$, $\theta_{x}$, and $\theta_{y}$ are set.

In the first step, the results of the static calculation can be used as the initial value for quasi-static calculations. In the second step, the quasi-static calculation with the following implementation algorithm is applied:

1. The contact angle obtained by static calculations is changed at both ends. Then, the large value of $\alpha_{i}$ is taken as the small value $\alpha_{o}$ and as the initial value of iteration.
2. If the nonlinear equation does not converge in the previous step, set the contact angle between the inner ring and the outer ring to change gradually until the equation converges.
3. The contact angle of the previous step is used as the initial contact angle of the next ball iteration until the contact angles of all balls are solved.
4. Considering obtained contact angles as the known values, the inner-ring balance relation is applied to solve the problem.
5. The remaining 36 + 5 nonlinear equations are solved by using the contact angle and the position of the inner ring as the initial iterative values.

## Simulation of rolling bearing runouts

By changing the position angle of each ball in the ball bearing and simulating the 5-DOF quasi-static model, the position ($x$,$y$, $z$,$\theta_{x}$,$\theta_{y}$) and its runout trajectory of the ball bearing's rotating center can be obtained.

The simulation was carried out in a number of cases. Specifically, the spherical diameter error and sphericity error and error factors of raceway sway, groove shape, and roundness were studied. The following parametric equation is introduced to describe the bearing runout

21 $$ \left\{ \begin{gathered} x(\theta ) = a\sin (\theta ) \hfill \\ y(\theta ) = b\cos (n\theta { + }\phi ). \hfill \\ \end{gathered} \right. $$

Equation (21) was applied to evaluate the radial runout. Moreover, the following equation was used to evaluate the axial runout:

22 $$ \left\{ \begin{gathered} x(\theta ) = a\sin (\theta ) \hfill \\ z(\theta ) = b\cos (n\theta { + }\phi ), \hfill \\ \end{gathered} \right. $$

where $a$ and $b$ are the values of two directions perpendicular to each other, $\theta$ is the position angle,$\phi { = }\pi /2$, and $n = 1$.

## Raceway error measurement

**Figure 3:**
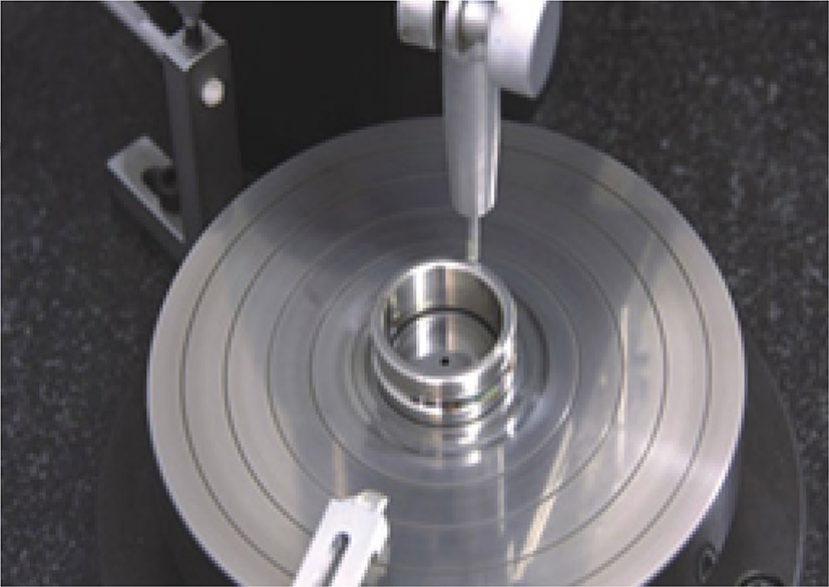
Photograph of raceway error measurement.

The correlation error was measured. The raceway errors are shown in Table 1 and the ball errors in Table 2. Figure 3 illustrates the raceway error test.

**Table 1:** Raceway error measurement.

	Max	Min	Max–Min
Roundness of inner raceway/μm	1.1	0.9	0.2
Roundness of outer raceway/μm	1.5	1.3	0.2
Groove shape of inner raceway/μm	1.3	1	0.2
Groove shape of outer raceway/μm	1.2	1	0.1
Inner side-sway/μm	1.7	1.5	0.2
Outer side-sway/μm	1.5	1.2	0.3

**Table 2:** Ball error measurement.

	Max	Min	Max–Min
Ball Diameter/mm	12.7004	12.7001	0.0003
Sphericity/μm	0.19	0.17	0.02

## Results of the rolling bearing runout

In this section, five sets of 7014 series bearing were considered as the simulation objects. Then, the average error of the raceway and ball was calculated to predict the bearing runout. Different assumptions were considered and simulations were performed accordingly.

**Figure 4:**
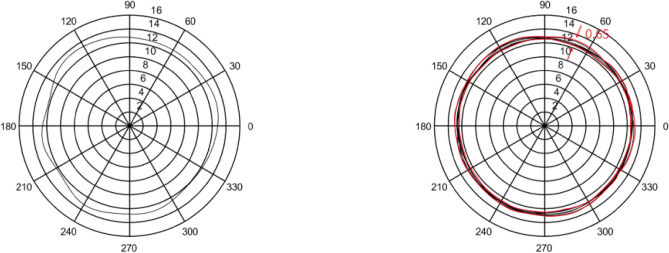
Distribution of the ball diameter error.

**Figure 5:**
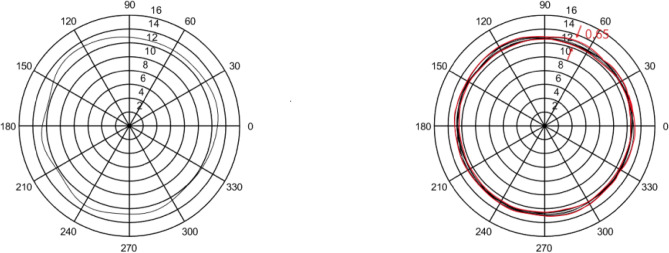
Distributions of the sphericity error.

**Figure 6:**
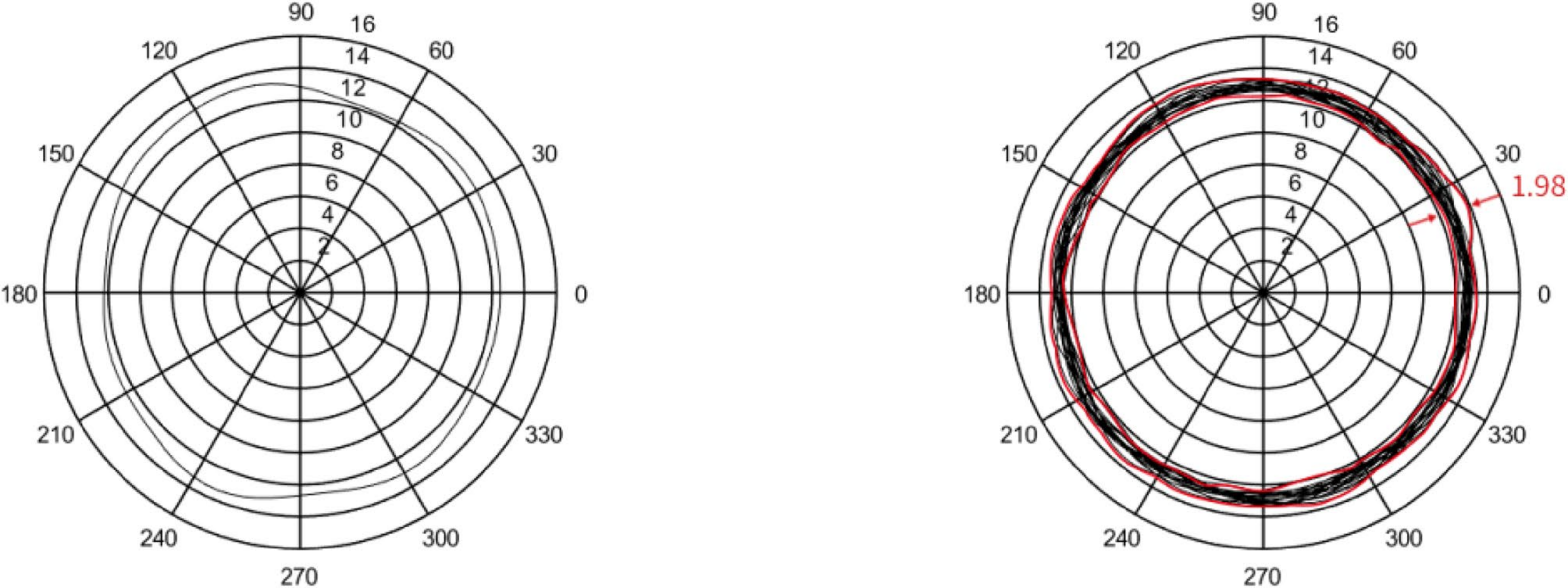
Distributions of the raceway side-sway error.

**Figure 7:**
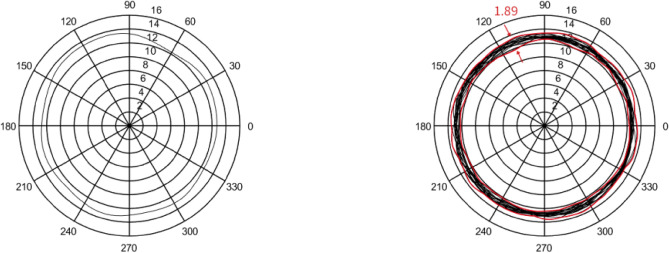
Roundness error of the raceway.

**Figure 8:**
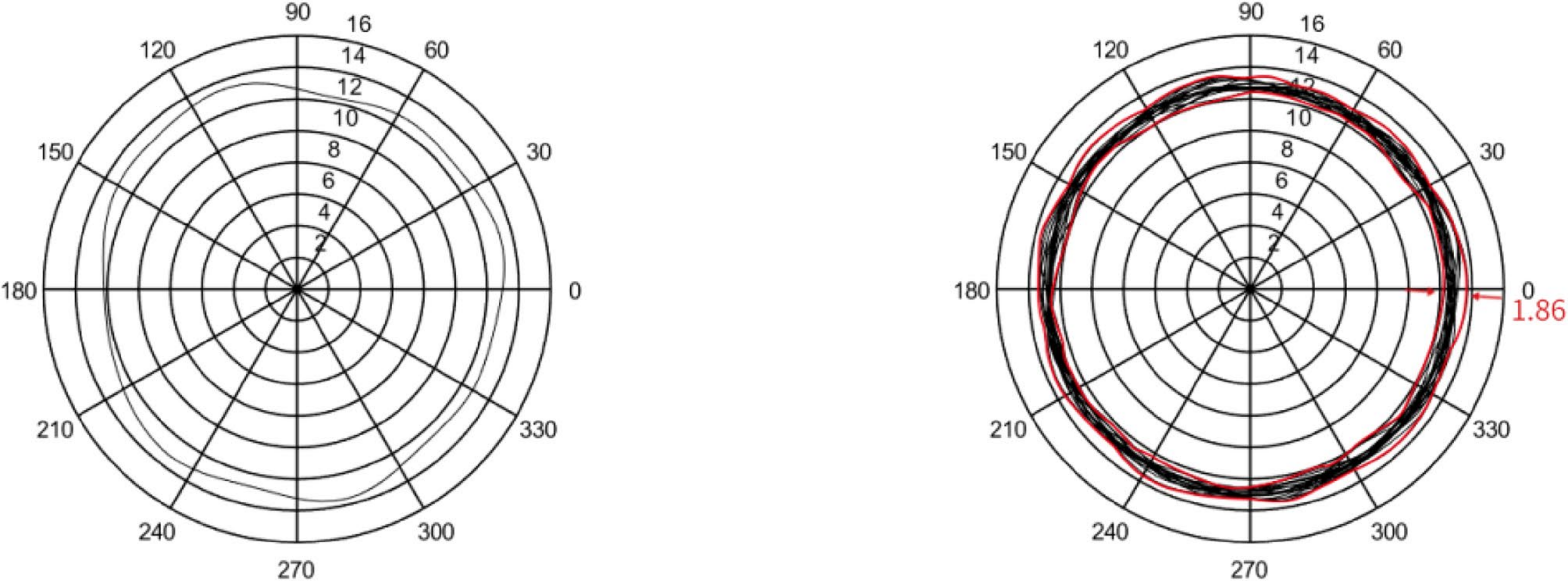
Groove shape error of the raceway.

**Figure 9:**
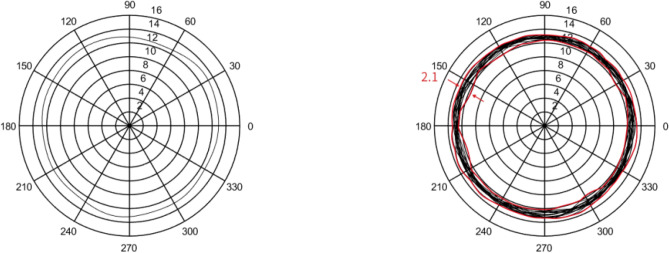
Simulation results of the steel ball raceway error.

1. In the simulation, it was assumed that the diameter error of nine steel balls is 0.40 μm, while other errors are considered as zero. The runout is defined as the distance between the maximum and minimum envelope circles. Figure 4 shows that the bearing radial runout is 0.55 μm.
2. In the simulation, it was assumed that nine steel balls have a sphericity error of 0.21 μm, while other errors are assumed to be zero. Moreover, the runout is defined as the distance between the maximum and minimum envelope circles. Figure 5 indicates that the bearing radial runout is 0.55 μm.
3. It was assumed that there was an error in the raceway roll. The rolling errors of the inner and outer rings were set to 1.6 and 1.7 μm, respectively. Moreover, other errors are assumed to be zero. Figure 6 shows that the bearing axial runout is 1.98 μm.
4. It is assumed that there is an error in raceway roundness. The roundness errors of the inner and outer rings were set to 1.5 and 1.6 μm, respectively. Moreover, other errors were assumed to be zero. Figure 7 indicates that the radial runout of the bearing is 1.89 μm.
5. It was assumed that error in the raceway groove shape is 3, 1.5, and 0 μm. Figure 8 shows that the bearing radial shaft is 1.86 μm.
6. It was assumed that both the ball and the raceway had errors. In this regard, the diameter error and sphericity errors of nine steel balls were set to 0.40 and 0.21 μm, respectively. Moreover, rolling errors of the inner and outer rings were set to 1.6 and 1.7 μm, respectively. The roundness errors of the inner and outer rings were set to 1.5 and 1.6 μm, respectively. Figure 9 shows that the radial shaft of the bearing was 2.1 μm.

## Bearing runout test

**Figure 10:**
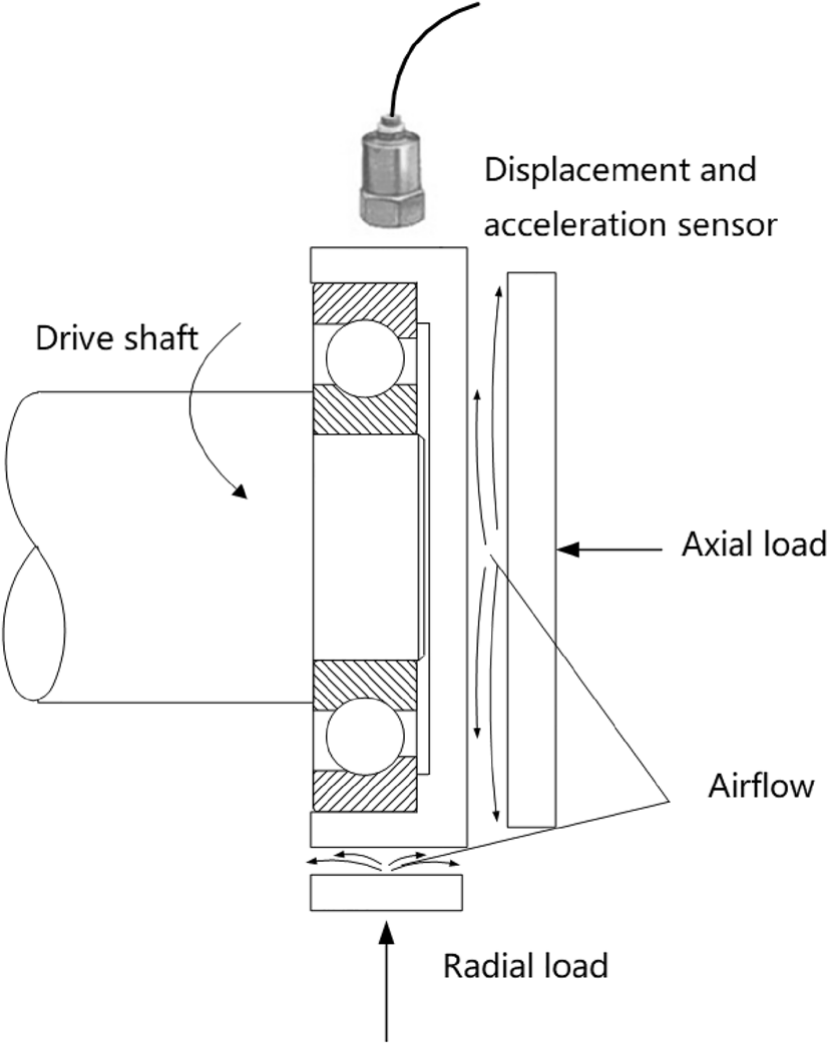
Schematic diagram of the bearing runout test bench.

The test bench uses the fixed outer-ring and rotating inner-ring measurement methods. The high-precision spindle drives the inner ring of the bearing in rotation, and the air bearing loads the outer ring of the test bearing axially and radially without contact. The displacement sensor and the triaxial acceleration sensor then each measure the displacement and the vibration acceleration of the outer ring of the bearing. Figure 10 illustrates the bearing runout accuracy test rig layout.

The experimental platform consists of hardware and software. The hardware part is composed of a marble base, a servo drive system, an air spindle support system, a precision mandrel, a bearing to be tested, an air loading module, and a measuring system. The measuring system includes a laser displacement sensor, a capacitive displacement sensor, an acceleration sensor, a signal acquisition instrument, and an industrial computer.

**Figure 11:**
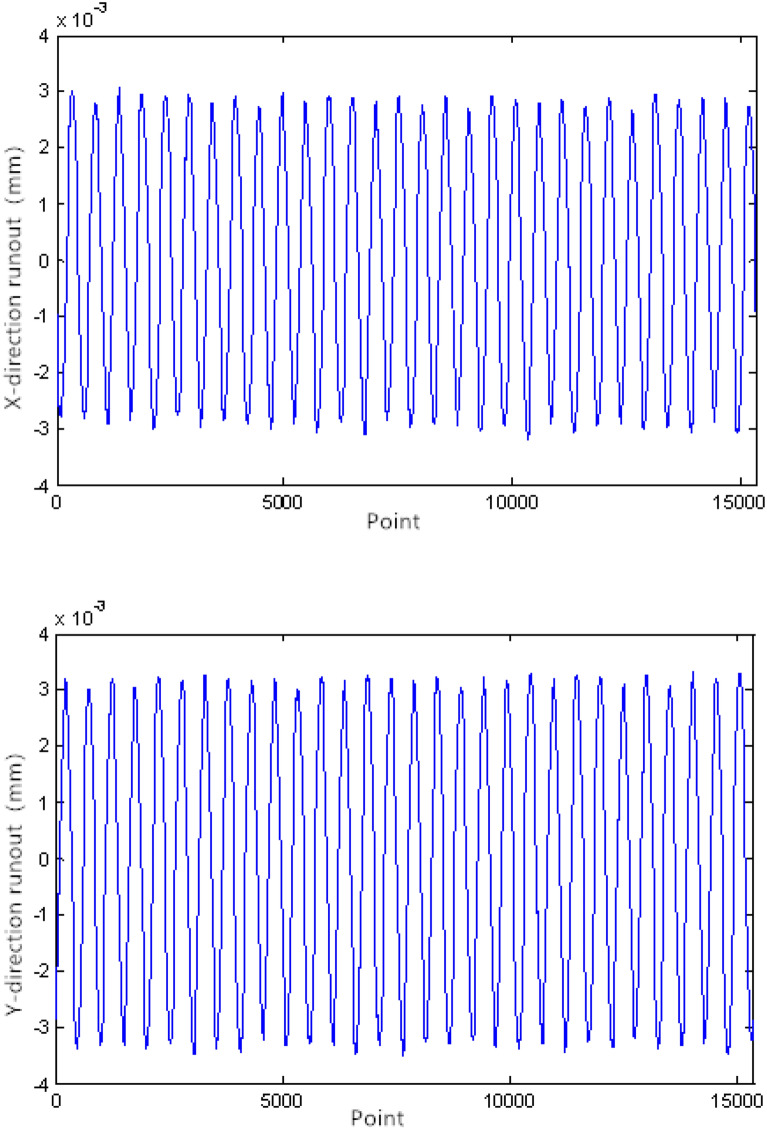
Runout signal.

The bearing rotation accuracy test was used to verify the bearing runout simulation. The bearing rotation accuracy test was completed under the condition that the axial force was 500N; the speed was set at 30 rpm, 60 rpm, 240 rpm, and 960 rpm alternately; the sampling frequency at 30 rpm was 256 Hz. When the speed was doubled, the sampling frequency doubled, and the number of sampling points was 15,359. The original runout signal measured at 30 rpm is shown in Fig. 11.

## Verification of the model

**Figure 12:**
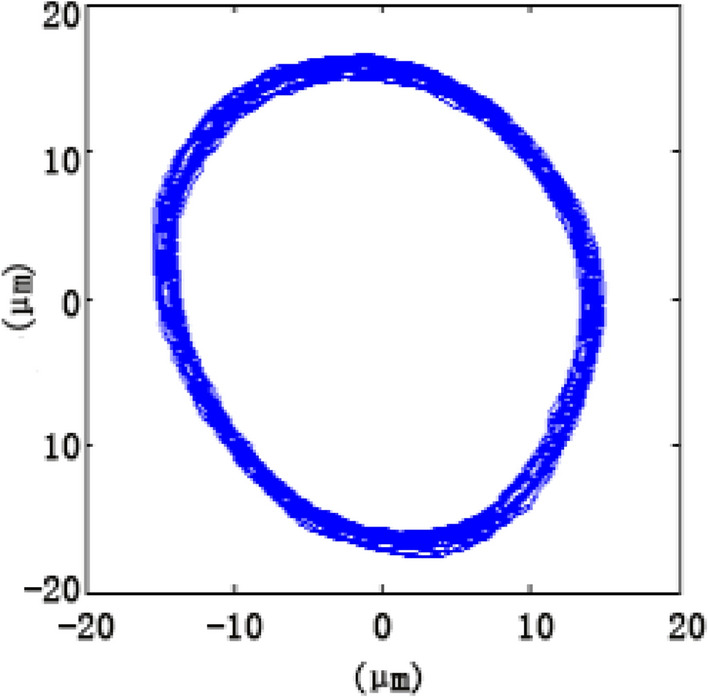
Bearing runout track.

The bearing rotation accuracy test was used to verify the bearing runout simulation. The accuracy of the quasi-static model of the rolling bearing was evaluated by comparing the obtained results from the simulation with that of the experiment. The runout track of 7014 series bearing was measured, as shown in Fig. 12. The measured radial runout of 7014 series bearing was 1.9 μm, indicating that with increasing rotating speed, the distributions of the bearing runout simulation and measurement were constant.

The relevant P2 accuracy raceway tolerances are given below. Local tolerances are given in Table 3 and foreign tolerances in Table 4.

**Table 3:** Local P2-grade bearing tolerance.

d/mm		Δdmp	Δds	Vdsp	Vdmp	Kia	Sd	Siab	ΔBs			VBs
				max	max	max	max	max				max
30	50	0	− 2.5	2.5	1.5	2.5	1.5	2.5	0	− 120	− 250	1.5
50	80	0	− 4	4	2	2.5	1.5	2.5	0	− 150	− 250	1.5

**Table 4:** SKF group P2-grade bearing tolerance.

d/mm		Δds		Vdp	Vdmp	ΔBs		ΔB1s		VBs	Kia	Sd	Sia
30	50	0	− 2.5	1.3	1	0	− 120	0	− 250	1.3	2.5	1.3	2.5
50	80	0	− 3.8	2	1.3	0	− 150	0	− 250	1.3	2.5	1.3	2.5

**Figure 13:**
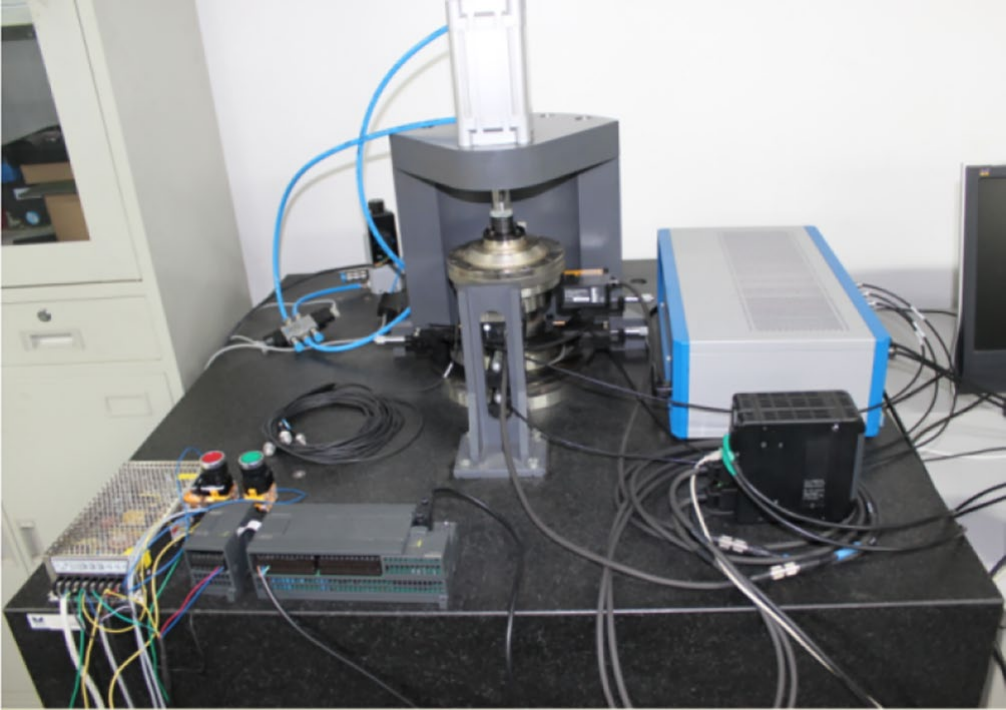
Test platform for measuring the bearing runout.

Figure 13 shows the bearing runout accuracy test rig.

## Rolling bearing error equalization

Rolling bearing error equalization effect refers to the rolling bearing operation process, due to the tolerance and error between the rolling body and raceway, will produce a self-adaptive equalization effect. Specifically, the rolling bearing rolling body and the raceway will occur between the relative rolling, rolling body will produce rolling track on the raceway. Due to the tolerance and error between the rolling body and the raceway, the rolling trajectory is not completely uniform, but there are certain fluctuations and variations.

This fluctuation and change of rolling trajectory will lead to uneven load distribution of rolling bearing, some rolling bodies bear larger load, while other rolling bodies bear smaller load. However, since the rolling bearing has a large number of rolling bodies and their relative positions to each other are also random, the load distribution of the rolling bearing as a whole will tend to be uniform. This adaptive homogenization effect can reduce the fatigue life and failure risk of rolling bearings and improve the reliability and service life of the bearings.

It should be noted that the error homogenization effect of rolling bearings is not completely homogenized, and there are still certain errors and fluctuations. Therefore, in some application scenarios with high requirements for bearing accuracy, such as high-speed operation or high-precision transmission systems, additional measures may be required to further reduce bearing errors and fluctuations to meet more stringent performance requirements.

### Definition of the rolling bearing error equalization coefficient

The radial homogenization coefficient can be defined as the ratio between the roundness error of the inner and outer rings and the radial runout of the bearing. The average coefficient of the radial error is an important index to measure the effect of error equalization. Generally, the average coefficient of radial error can be expressed as follows:

23 $$ \delta_{x} = \frac{\max (\left| X \right|)}{{\left| E \right|}}, $$

24 $$ E = E_{1} + E_{2} , $$

where $X$ denotes the radial runout of the rolling bearing and $E$ is the sum of the inner and outer raceway waviness. Moreover, $E_{1}$ and $E_{2}$ are the waviness of the inner and outer rings, respectively.

The average coefficient of the axial error can be defined as the ratio of the inner and outer ringside sway error to bearing axial runout. The average coefficient of the axial error, which is defined in Eq. (25), can be considered as an important index to measure the effect of error equalization.

25 $$ \delta_{z} = \frac{\max (\left| Z \right|)}{{\left| E \right|}}, $$

26 $$ E = E_{3} + E_{4} , $$

where $Z$ is the axial runout of the rolling bearing, and $E$ denotes the sum of the inner and outer ring raceway sway or the sum of the inner and outer ring raceway groove shape. Moreover, $E_{3}$ and $E_{4}$ denote the inner and outer ring side-sways or groove shapes, respectively.

### Analyzing error homogenization results of the rolling bearing

**Figure 14:**
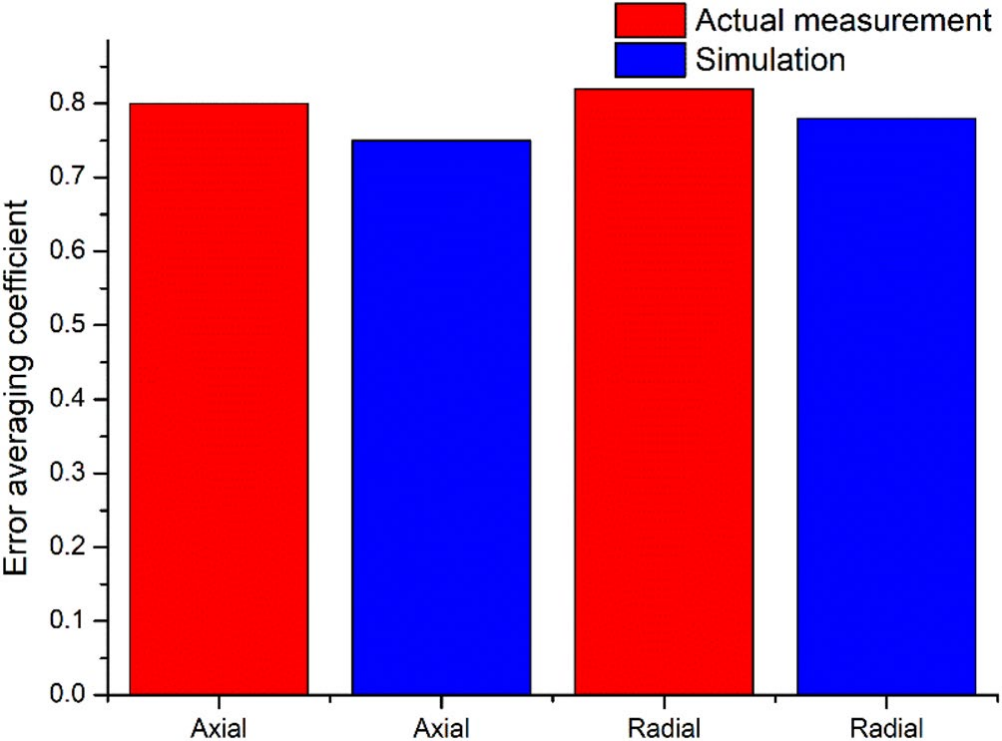
Comparison of error equalization effect.

Figure 14 shows that the homogenization capability of the radial runout error is higher than that of the axial runout error. It is observed that the average coefficient of simulation error is smaller than that of the actual measurement error, indicating that the average impact of simulation error is higher than that of the actual measurement error.

## Raceway tolerance design derived from the runout model

**Figure 15:**
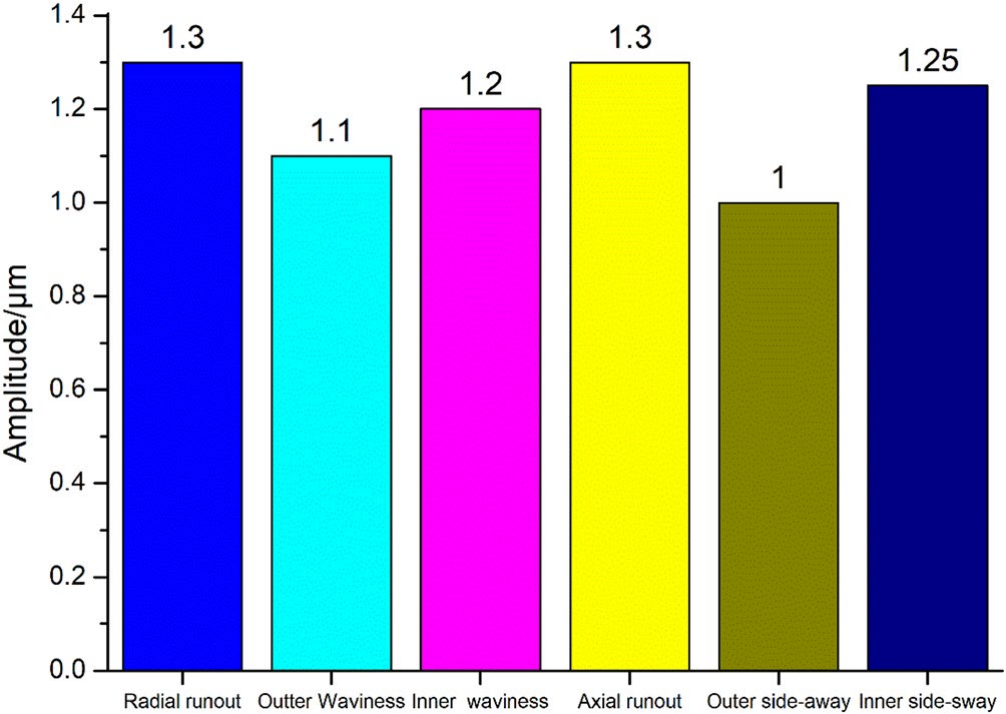
Economic design of raceway tolerance for 7014 P2 bearing.

Studies show that radial and axial runouts are the main indices to evaluate the bearing accuracy. To manufacture P2 level rolling bearings, it is necessary to establish the technical requirements for the P2 level bearing raceway in theory. The manufacturing accuracy of the P2 bearing raceway is not determined by the high values; therefore, it is necessary to propose a quantitative index for the raceway accuracy. Through investigating the error equalization effect of the rolling bearing, the accuracy requirements of the P2 level bearing raceway were obtained. Since the average error effect of radial and axial runouts is between 0.6 and 0.8, the radial and axial runouts of 7014 P2 rolling bearings are 1.3 μm. These results are shown in Fig. 15. From Fig. 14 it can be observed that the requirement of the side sway and waviness of the outer ring raceway was slightly lower than that of the inner ring raceway by an order of magnitude. Moreover, it was found that the sum of the inner and outer rings raceway errors varied within the range of 0.6–0.8 of the bearing runout error by an order of magnitude.

## Conclusions

The following conclusions can be drawn.

1. The rolling bearings showed an error homogenization effect. Based on the obtained results of the rotation accuracy, the difference in the raceway accuracy was analyzed, indicating that the radial runout is less than the roundness error of the inner and outer raceways, and the axial runout is less than the side-sway error of the inner and outer raceways. A 5-DOF quasi-static model of the ball bearing was established. The raceway roundness and groove shape were characterized by radial and axial waviness. The error homogenization coefficient of the rolling bearing was defined, and an error equalization effect in the rolling bearing was proven.
2. When the average coefficient of raceway error is between 0.6 and 0.8, the theoretical error equalization yields the best results, while the actual error equalization effect is the worst. Moreover, the simulation error homogenization effect is between theory and practice.
3. According to the requirement of the P2 level bearing runout, the error range of the bearing raceway sway and roundness in theory was derived, indicating that the side sway and waviness of the outer-ring raceway were slightly lower than those of the inner-ring raceway by an order of magnitude. The sum of the inner-ring raceway error and outer-ring raceway error was still 0.6–0.8 times that of the bearing runout error by an order of magnitude.

## Data Availability

The data that support the findings of this study are available from [Wenhan Cao] but restrictions apply to the availability of these data, which were used under license for the current study, and so are not publicly available. Data are however available from the corresponding authors upon reasonable request and with permission of [Wenhan Cao].
